# The ECM: To Scaffold, or Not to Scaffold, That Is the Question

**DOI:** 10.3390/ijms222312690

**Published:** 2021-11-24

**Authors:** Jonard Corpuz Valdoz, Benjamin C. Johnson, Dallin J. Jacobs, Nicholas A. Franks, Ethan L. Dodson, Cecilia Sanders, Collin G. Cribbs, Pam M. Van Ry

**Affiliations:** Department of Chemistry and Biochemistry, Brigham Young University, Provo, UT 84602, USA; jcvaldoz@byu.edu (J.C.V.); benj821@byu.edu (B.C.J.); djjacobs@byu.edu (D.J.J.); naf425@byu.edu (N.A.F.); ethanld@byu.edu (E.L.D.); cecili23@byu.edu (C.S.); ccribbs@byu.edu (C.G.C.)

**Keywords:** tissue engineering, spheroids, organoids, scaffold-based, scaffold-free, ECM, hydrogel

## Abstract

The extracellular matrix (ECM) has pleiotropic effects, ranging from cell adhesion to cell survival. In tissue engineering, the use of ECM and ECM-like scaffolds has separated the field into two distinct areas—scaffold-based and scaffold-free. Scaffold-free techniques are used in creating reproducible cell aggregates which have massive potential for high-throughput, reproducible drug screening and disease modeling. Though, the lack of ECM prevents certain cells from surviving and proliferating. Thus, tissue engineers use scaffolds to mimic the native ECM and produce organotypic models which show more reliability in disease modeling. However, scaffold-based techniques come at a trade-off of reproducibility and throughput. To bridge the tissue engineering dichotomy, we posit that finding novel ways to incorporate the ECM in scaffold-free cultures can synergize these two disparate techniques.

## 1. Introduction

Tissue engineering is an interdisciplinary field with a goal of creating biomimetic tissues and organs. Modern tissue engineering has been applied to create a variety of tissues and organs, from minute aggregates of human cells [1] to large-scale bioengineered cartilage [2]. The potential impacts of tissue engineering include tissue and organ replacement, disease modeling, high-throughput drug screening, and personalized medicine. Tissue engineering attempts to regenerate whole organs, and it partially alleviates the shortage of certain organs available for donation [3]. The gap between the number of patients awaiting transplantation and the number receiving organs continues to rise because of the increases in donor organ shortages [4]. There is also a need for better in vitro organ models to recapitulate the human response to disease or novel drugs. Currently, 80% of drugs tested fail clinical trials, partly due to drug response differences between animal and human systems [5]. Tissue-engineered organ models represent human organ responses with higher fidelity and have the potential to improve the short coming of current models [5]. Moreover, there is a great potential for tissue engineering in creating patient-specific drug screening platforms [6,7]. These platforms can predict variability in responses to therapies, allowing for personalized treatment of heterogeneous diseases, such as cancer [1].

In tissue engineering, a dichotomy of somewhat opposing techniques is being employed—scaffold-based or scaffold-free [8]. The basis of this dichotomy is whether solid scaffolding material is essential to support three-dimensional cell growth. In scaffold-based tissue engineering, a very common material used is the extracellular matrix (ECM), the non-cellular component of tissues and organs. The composition and mechanical properties of the ECM affect many cell functions, including cell anchorage [9], morphogenesis [10], signaling [11], and survival [12]. Associated with the ECM are a variety of structural proteins (collagens, elastin, laminin, and fibronectin), growth factors, and glycans (such as hyaluronic acid), small leucine-rich proteoglycans (SLRPs), modular proteoglycans, and cell-surface proteoglycans [13].

Here, we will explore the central roles of the ECM in biological adhesion, receptor signaling, cell survival, and morphogenesis that make it the go-to material for tissue engineering. We will also explore the recent technological advancements in mimicking the ECM via synthetic polymers. Moreover, we will explore the tissue engineering dichotomy and the pros and cons of each technology. We will illustrate novel methods of incorporating the ECM or other scaffolds into scaffold-free technologies leading to a hybrid culture system. This hybrid system shows improved reproducibility of tissue-engineered materials and replication of organ-like features in aggregates.

## 2. The Extracellular Matrix

**Figure 1:**
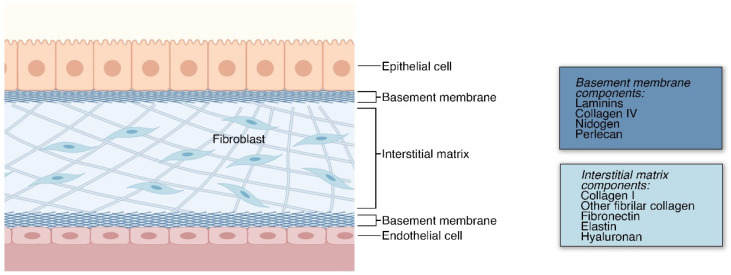
Organ stratification by cell type and ECM type. In most organs, layering of cells and the ECM is observed. The outer most layer comprises of the epithelial cells which have specialized functions specific to the organs. These epithelial cells are anchored to the organ via a basement membrane which has a plethora of functions. Below this is the IM which houses the fibroblasts and fibrillar ECM proteins responsible for structural support. Bordering the IM is another layer of basement membrane which is associated with the blood vessel components, such as the endothelial cells. Both ECM types are comprised of a different set of ECM proteins. The IM, due to its functions in structure of the organs and tissues, is comprised of fibrillar ECM, such as fibronectin and collagen I. Whereas, the basement membrane is comprised of laminins and other proteins that are essential for epithelial and endothelial cell homeostasis.

The ECM can be divided into two categories: basement membrane (BM) and interstitial matrix (IM). The BM is a layer of ECM in contact with all epithelium and endothelium, which separates tissues within the body [14]. In contrast, the IM includes all other ECM between cells in tissues [13]. In heart tissue, it has been shown that rapid ECM synthesis in embryonic development precedes cell proliferation and that the stratification of the ECM begins in late embryonic development and continues into postnatal life [15]. Although much is yet to be determined about the biophysical development of the ECM, time-lapse tracking of ECM markers has revealed that it is dynamic throughout morphogenesis, using both cell-autonomous displacements and large-scale tissue movements [16,17]. The basic organ and ECM type stratification is summarized in Figure 1.

The basement membrane (BM) is a specialized ECM that forms in thin, compact layers of type IV collagen, laminins, nidogen, perlecan, and other proteoglycans. There is abundant research showing that this ECM surrounds animal epithelial and endothelial tissues where it is produced [18,19]. Findings also show that keratinocytes and fibroblasts also produce BM components [20]. Moreover, knockout studies of collagen IV show that the BM is initially formed primarily with laminins and later includes collagen IV [21]. Though, this process of stratification and organization is not well understood.

The BM is widely known to provide structural support in tissues and also plays important roles in cell behavior, including cell adhesion, migration, and compartmentalization [14,22]. Moreover, several constituents of the BM serve different functions. In the BM, type IV collagen forms networks through covalent disulfide and sulfonimine bonds [23]. This network of type IV collagen provides mechanical support and contributes to the tensile strength of the basement membrane [24]. These networks act as a substrate which cells adhere to, and are present at cell-binding sites [25]. According to one study, collagen IV knockout is mouse embryonic lethal at embryonic stage E11.5, suggesting that it is indispensable during morphogenesis [21]. There is evidence that basement membrane laminins play a vital role in angiogenesis and skeletal muscle health [26]. Moreover, one study showed that the removal of laminin from the BM in neuromuscular tissues impaired adhesion to BM which resulted in the detachment of motorneuronal terminals [27]. As cells adhere to the BM, it becomes a scaffold for cell signaling [28]. Lastly, perlecan and other proteoglycans in the BM can then bind to various growth factors and incite cell differentiation, migration, and other cell functions [28,29].

In the IM, fibroblasts and myofibroblasts, and a variety of other cells, secrete fibrous matrix proteins and glycans, and constantly remodel and maintain the ECM [30]. The IM is primarily composed of proteoglycans complexed in the form of a hydrated gel [31]. In the IM, collagen is the most abundant fibrous protein, with type I collagen being the most prevalent for structural support [30]. Another key component of the IM, hyaluronan, is essential for regulating turgidity in the interstitial fluid, providing an elegant modulator of transport rates between tissues [32] which is exploited by tumors to resist drug delivery [33,34]. The IM participates in signaling as much as it does in structure formation; a dominant proposed model is binding-mediated hindered diffusion [35]. This model shows a repeatedly observed theme of extracellular molecules occupying signaling molecules, with processes that slow their transport. As well as hindering, IM components occasionally facilitate diffusion [32], such as in exosomal transport of upregulated proteins in cardiac tissue [36] and in transport of miRNA vesicles [37], although the extracellular support mechanisms of these vesicles remains elusive.

Aberrant ECM production and regulation contributes to many diseases, including fibrosis, cancer, diabetes, and myopathies. In idiopathic pulmonary fibrosis, the scarring of lungs is caused, in part, by an excessive production of collagen I, fibronectin, and other ECM components. In fibrosis, ECM accumulation is promoted by increased activity of yes-associated protein 1 (YAP)/transcriptional coactivator with PDZ-binding motif (TAZ), transforming growth factor beta (TGFβ), and wingless/Int (WNT) [38,39,40,41,42]. In breast cancer, the mechanism for stiffness has been described as a cross-linking of collagen caused by tumor-associated macrophages [43]. Scarring found in various diabetic tissues, including the retina [44] and the kidney [45], is caused, respectively, by advanced glycation end-product induction of ECM protein crosslinking and by an excessive deposition of ECM proteins in the basement membrane, both of which lead to tissue stiffening. Lastly, mutations in ECM and ECM-related genes produce myopathies and muscular dystrophies. Examples of loss of function in ECM components could be observed in laminin α2-related congenital muscular dystrophy (LAMA2-CMD) and collagen VI myopathies [46,47,48]. The diverse modifications in ECM seen in pathology support the idea that it is not just the mere amount of protein that determines the normal function of the ECM, but also the dynamic remodeling [30].

### 2.1. ECM in Structural Support and Anchorage

Variability in strength, elasticity, and other mechanical properties from tissue to tissue within an animal can be attributed to different compositions of collagen and elastin, structural differences [49], and the presence of various proteoglycans [50]. As observed by decellularizing various tissues, the ECM alone provides much of the macrostructural support to maintain the shape of organs and bodies [51]. Its high tensile strength is largely due to its collagen composition [52]. Collagen fibrils extracted from rat patellar tendon were found to have a relatively high tensile strength of 71 ± 23 MPa [53]. While the ECM demonstrates high tensile strength in some tissues, it shows high elasticity in others [54]. The protein elastin and other proteoglycans add to collagen’s native elasticity [54]. The Young’s modulus of elastin fibers was found to be 1 MPa, whereas the Young’s modulus in collagen fibers was found to be between 250–400 MPa [55]. Compositing collagen and elastin yields mechanical properties dependent on the percent composition of each. Beenakker et al. reported that films of collagen combined with increasing percentages of insoluble elastin decreases the overall stiffness and increases the overall strain to failure of that film [56].

The ECM also has a well-known role in cell anchorage. It operates as a scaffold on which cells can build and function [13]. On the porous, fibrous microstructure of the ECM, cells receive nutrients where they may otherwise be too densely packed for fluid and molecular flow [57]. The ECM also allows for cell anchorage through specialized, cell-adherent structures. Cells use proteins known as integrins, selectins, and cadherins to attach themselves to the ECM proteins, such as fibronectin and laminin [54]. Fibronectin primarily regulates the attachment of cells to the ECM [13]. As fibronectin is stretched, several integrin-binding sites are exposed, which initiates a cascade of cell adhesion and alignment events [13,54]. Laminins are also associated with cell anchorage. These proteins have integrin-binding sites and are also known to bind to glycans associated with the cytoskeleton [58].

### 2.2. ECM in Receptor Signaling

**Figure 2:**
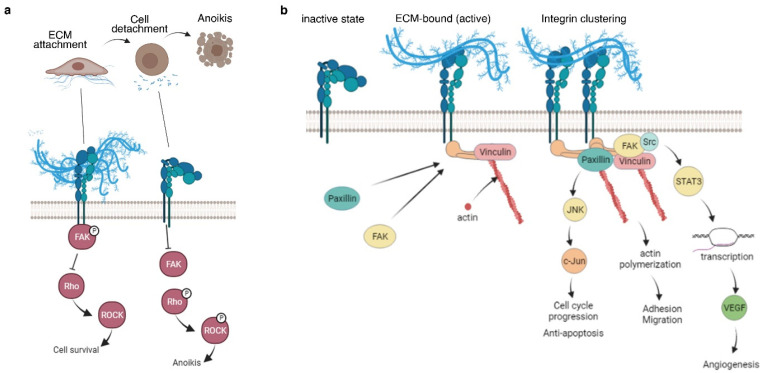
ECM–integrin signaling. The most established transduction pathway for ECM interactions to the cell is through integrin signaling. (**a**) During cell detachment, activation of ROCK signaling leads to anoikis [74]. Thus, inhibition of ROCK has been beneficial in tissue engineering to prevent cell death during cell dissociation, especially in stem cells [75]. (**b**) Integrin signaling not only prevents cell death but also activates a plethora of signaling cascades in the cell [66,67,76,77]. Some of the downstream effects of integrin–ECM interactions include cell cycle progression leading to cell survival and proliferation, promotion of cell polarity, migration, adhesion, and transcriptional control of key pro-angiogenic factors (such as VEGF).

The interaction between ECM proteins and integrins plays essential roles in cell signaling [59]. Integrins are heterodimeric transmembrane receptors that bind to ECM proteins as their ligands [60]. Thus, integrins can also respond to either changes in ECM composition or mechanical forces [61]. There are a variety of integrins, which have receptors for specific ECM proteins, including fibronectin and collagen [59]. Depending on the composition of these ECM proteins, integrins initiate a signaling cascade to regulate proliferation, survival, and migration [62]. Integrins are also mechanotransductors, which means they respond to mechanical forces with chemical stimuli [63]. An increase in ECM stiffness leads to a loss in functional structure and increased invasiveness in tumors [64]. The exact composition of particular integrins is unique to different tissue which helps regulate growth in particular organs [64,65]. Although not covered extensively in this review, some of the key ECM-integrin signaling pathways are illustrated in Figure 2 [60,62,63,65,66,67,68,69,70].

The ECM has also been shown to contribute to growth factor (GF) signaling pathway. ECM proteoglycans selectively bind GFs, which allows the ECM to function like a reservoir of GFs [11]. As the ECM is degraded, the bound GFs are released to the surrounding area, stimulating growth. Furthermore, the ECM’s ability to bind GFs creates a morphogen gradient necessary during embryonic development. Aside from the release of bound GFs, ECM degradation products have cytokine and chemokine-mimetic roles [71]. During ECM remodeling and degradation, a variety of ECM proteins, including collagens and laminins, are precursors to signaling molecules that have roles in very diverse signaling processes [72]. An example is the collagen I-derived proteolytic fragment Pro-Gly-Pro (PGP), which is a neutrophil chemoattractant acting as a ligand to CXC chemokine receptors 1 and 2 on neutrophils [73]. Even in solid form or without remodeling, the ECM can contribute to GF signaling pathways as certain ECM domains can act as cofactors to GF receptors [11,72]. Through this mechanism, the tissue-specific ECM composition also modulates a tissue’s sensitivity to GFs.

#### 2.2.1. ECM in Morphogenesis

ECM contributes to morphogenesis, the biological process of proper tissue and organ formation. Because of this, ECM-based scaffolds are often used in stem cell differentiation to create organoids. Particularly, cell differentiation occurs in monkey blastocyst stem cells with the supplementation of either solid or solubilized BM extract [78]. Aside from this, feeder-free maintenance of human embryonic stem cells is achieved when cultured on matrigel in a conditioned media [79].

The variety of structural proteins and signaling molecules in ECM provides the necessary combination of rigidity and elasticity which help sculpt tissues into functional organ structures. Several lines of research show that ECM contributes to branching during organ formation [78]. Studies genetically altering the components of the ECM in animal models show that fibronectin, collagen, and laminin all play roles in epithelial branching during organogenesis [72]. Additionally, mammary gland branching is enhanced in collagen I gels [80], but inhibited in laminin-rich basement membrane gels [81], which suggests the varying roles of different proteins during branching morphogenesis [82]. There is also a correlation between ECM stiffness and cell differentiation. In a recent report by Garreta and colleagues, differentiation of kidney organoids from induced pluripotent stem cells (iPSCs) is accelerated by 3D soft hydrogels [83]. Moreover, TGFβ-dependent smooth muscle differentiation from mesenchymal stem cells is enhanced by matrix stiffness [84]. Thus, precise control of matrix stiffness alone has a significant effect on cell differentiation and morphogenesis.

ECM has also been connected to increased angiogenesis, the development of blood vessels. Angiogenesis begins with the degradation of capillary basement membrane [85]. Eventually endothelial cells invade the ECM and begin to form immature capillary structures. The correct composition of ECM proteins is vital for proper angiogenesis to occur. Elastin, collagen, and fibronectin contribute to angiogenesis through a combination of structural support and cell-to-cell signaling. Elastin is a major part of the ECM scaffold that helps with blood vessel composition and signaling [86]. Elastin plays a major role in maintaining the proper blood vessel structure by preventing vascular occlusion [87]. Elastin is also proposed as a signaling molecule that controls VSMC migration and cell proliferation [88]. However, the exact elastin pathways for these signals still remain unknown. Fibronectin is another ECM protein that is essential to mechanical and signaling functions [89]. Fibronectin supports angiogenesis by preventing aortic rupture [90] in order to determine the polarity of cells during angiogenesis [89]. Collagen regulates migration adhesion and proliferation by binding to β1 integrins and discoidin domain receptors [91].

#### 2.2.2. ECM in Proliferation

Another established function of the ECM is the regulation of cell survival and proliferation. Integrin receptors not only provide a physical connection of cells to ECM, but also a signaling link that regulates upstream and downstream pathways [92]. It is well established that proliferation is dependent on attachment and growth factors delivered from the ECM reservoir [11]. Gospodarowicz et al. showed that bovine granulosa and adrenal cortex cells maintained on a plastic dish and seeded at low concentrations did not proliferate; however, cells maintained in the same serum and seeded at the same concentration proliferated when the plate was lined with decelullarized ECM [93].

Growth factor receptors are regulated by integrin-mediated cell adhesion [70]. Thus, when integrins are not bound to ligands, such as fibronectin, vitronectin, collagen, or laminin, the signaling cascade is interrupted, and some cell types experience decreased proliferation. ECM binding to integrins promotes a stronger extracellular signal-regulated kinases (ERK) signaling response than integrin clustering alone [94]. After activation, ERK is able to pass into the nucleus and phosphorylate transcription factors [95], enabling the transcription of genes affecting growth, proliferation, differentiation, and survival [96]. Additionally, because the ECM acts as a reservoir of growth factors, ECM degradation leads to the release of pro-mitogenic GFs [97].

#### 2.2.3. ECM in Cell Survival and Cell Death

The ECM has established roles in the control of cell survival and cell death. In fact, many types of cells undergo apoptosis when deprived of attachment to the ECM, a process known as “anoikis” [98]. In ECM-anchored cells, focal adhesion kinase (FAK) acts as an anoikic suppressor when activated by integrins [68]. Loss of the cell–matrix contact leads to a gap in this anoikis suppressor pathway. In anoikis, the activation of caspases initiated pathways leads to rampant endonuclease activity, DNA breakdown, and blebbing, resulting in cell death [86]. This process can initiate very rapidly when cells lose adherence. Grossman et al. found that, in unaltered human intestinal epithelial cells, caspases 2 and 9 were activated within 15 min of inducing non-attachment, causing activation of downstream caspases within an hour [99]. One of the downstream caspases, caspase 3, mediates cleavage of DNA fragmentation factor, leading to the breakdown of DNA [99]. ROCK 1, a downstream effector of the integrin signaling, has been implicated in contraction of cells and membrane blebbing observed in anoikic cells [100]. In tissue engineering, blocking the activity of ROCK via Y-27632 suppresses anoikis during cell dissociation in sensitive cells, such as embryonic stem cells [75].

Another pathway of non-attachment-induced cell death exists apart from anoikis. In caspase-independent cell death, metabolic changes occur, resulting from the lack of integrin binding. Schafer et al. found that matrix detachment causes a decrease in cellular ATP in epithelial cells due to a loss of glucose transport [101]. This occurred even in the presence of anoikis suppressors. They found that a reduction in glucose levels preceded an increase in reactive oxygen species (ROS) and a reduction in glutathione. These ROS generated from detachment were found to inhibit fatty acid oxidation (FAO), which supplies ATP in the absence of glucose. These results show a pathway by which the death of detached cells is achieved by nutrient deprivation, even in anoikis-inhibited cells.

### 2.3. Synthetic Materials as ECM Biomimetic

Whether from mechanical or biochemical sources, cells must receive proper environmental cues for proliferation, survival, and differentiation. In scaffold-based tissue engineering, biomaterials are incorporated into cell cultures to provide these signals. In tissue engineering, the most commonly used BM-derived biomaterial is the basement membrane extract (BME), including products under tradenames Matrigel, Cultrex, and Geltrex. BME often originates from murine sarcoma and exhibits features of the BM including high levels of collagen IV and laminins [102,103]. BME has since been used to promote cell differentiation and myotube development [103,104]. BME also provides a scaffold for organoid and spheroid development [105]. However, BME does have some deficiencies. The composition of BME varies from batch to batch due to its animal cell-derived nature, does not allow for controlled modifications, and contains antigens, precluding clinical application of BME-derived tissues [106].

To answer the problems of variability in animal-derived ECM, synthetic biomaterials have been developed. In this aspect, a recent review by Aisenbrey and Murphy elaborated on the several alternatives to BME in PSC maintenance, stem cell differentiation, in vivo tissue regeneration, organoid assembly, and disease modeling [107]. Many functions of the ECM—including structure, elasticity, biocompatibility, and bioactivity—can be replicated in cell culture with the presence of synthetic biomaterials, most commonly hydrogels. Hydrogels are 3D cross-linked polymer networks that absorb and retain large amounts of water. They are widely used in biomedical and tissue engineering applications because of their tunable properties and functionalities, as well as their relatively simple fabrication methods [108]. Although hydrogels can be inherently beneficial to 3D cell culture simply due to their elasticity and structural integrity, recent research aims to functionalize synthetic hydrogels to make them bioactive and to promote receptor signaling, morphogenesis, and cell survival, similar to the ECM.

Common synthetic polymer backbones used in tissue engineering are illustrated in Table 1. Cross-linkable polymers, such as poly(ethylene glycol) (PEG), poly(vinyl alcohol) (PVA), and poly(2-hydroxyethyl methacrylate) (PHEMA), can form an elastic, porous gel which mimics the mechanical properties of the ECM [109]. Synthetic hydrogels are ideal scaffolds for tissue engineering because their material properties can be tailored for desired outcomes. For example, research shows that variation in the elasticity of polyacrylamide hydrogel substrates affects the differentiation of mesenchymal stem cells via mechanotransduction [110]. Additionally, some hydrogels, such as PEG, can be fabricated with especially high porosity, resulting in the hydrogel acting as a reservoir of diffusible solutions with bioactive factors necessary for drug delivery [111].

Bioinert hydrogels alone provide only scaffolding to cells, clearly fulfilling just one function of the ECM; however, adding certain peptides into porous hydrogels can create an environment for cells to receive other components endogenously found in the ECM. For instance, cells require attachment to an adhesive substrate for survival; without this attachment, cells undergo anoikis. Fibronectin in the ECM meets this need because it contains RGD peptide motifs, to which cells can adhere via integrin [112]. In an ECM-mimetic hydrogel, RGD sequences on PEGMA (-monoacrylate) can be copolymerized with PEGDA (-diacrylate) molecules to achieve an adherable hydrogel surface for cells [113]. By blending, copolymerizing, or post-polymerizing hydrogels with RGD peptides, hydrogel adhesiveness has shown to promote cell survival [114], differentiation [115], and angiogenesis [116]. Another synthetic polymer that shows potency is polyamidoamine (PAMAM). Kim and Kinooka showed that surface functionalizing tissue culture plates with PAMAM dendrimers leads to increased cell migration and generation-dependent differentiation of iPSCs, single-generation dendrimer leads to maintenance, while fifth generation dendrimer promotes differentiation towards endodermal lineage [117].

**Table 1:** Common synthetic polymers backbones used in tissue engineering as ECM mimics.

Backbone	Structure	Citation
PEG	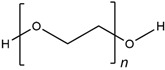	Wake, et al., 1996 [118] Hern and Hubbell, 1998 [119] Namba, et al., 2009 [120]
PVA	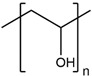	Wake, et al., 1995 [121] Annabi, et al., 2013 [109]
PHEMA	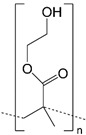	Flynn, et al., 2003 [122] Annabi, et al., 2013 [109]
PAMAM	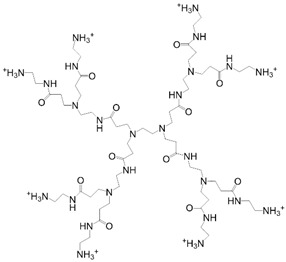	Kawase, et al., 1999 [123] Kim and Kino-oka, 2014 [117]
Dextran	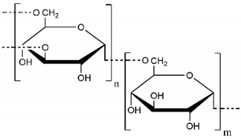	Chupa, et al., 2000 [124] Möller, et al., 2007 [125] Liu, et al., 2021 [116]

To replicate the pleiotropic functions of the ECM more faithfully, hydrogels need to be functionalized with a variety of bioactive factors [126]. Often, peptide sequences that have bioactive roles are used in functionalization and these are illustrated in Table 2. Early work shows functionalization by both cell adherent RGD and protease-sensitive PQ which then mimics both the adhesion and degradation remodeling of the ECM [127]. In this aspect, further modulation of peptide cleavage sequences is important. Previous research by Patterson et al. illustrated that PEG hydrogels functionalized with GPQGIWG or VPMSMRGG peptides, respectively, led to slower or faster cleavage rates by proteolytic enzymes MMP1 and MMP2, promoting cell migration [128]. In a recent report, dextran methacrylate (DexMA) and vinyl sulfone (DexVS) functionalized with an array of adhesive peptides and crosslinked with MMP-sensitive dicysteine peptides has a robust effect in angiogenic sprouting, leading to lumen formation [116]. Aside from these, several biomaterials show capability for controlled release of various substances, including growth factors and morphogens [129], oxygen [130], and drugs [131] among others. In summary, there is abundant research showing that functionalized synthetic biomaterials can mimic some features of the ECM, including degradation, cell adhesion, and controlled release, which has led to their prevalent use in tissue engineering.

## 3. The Tissue Engineering Dichotomy

**Figure 3:**
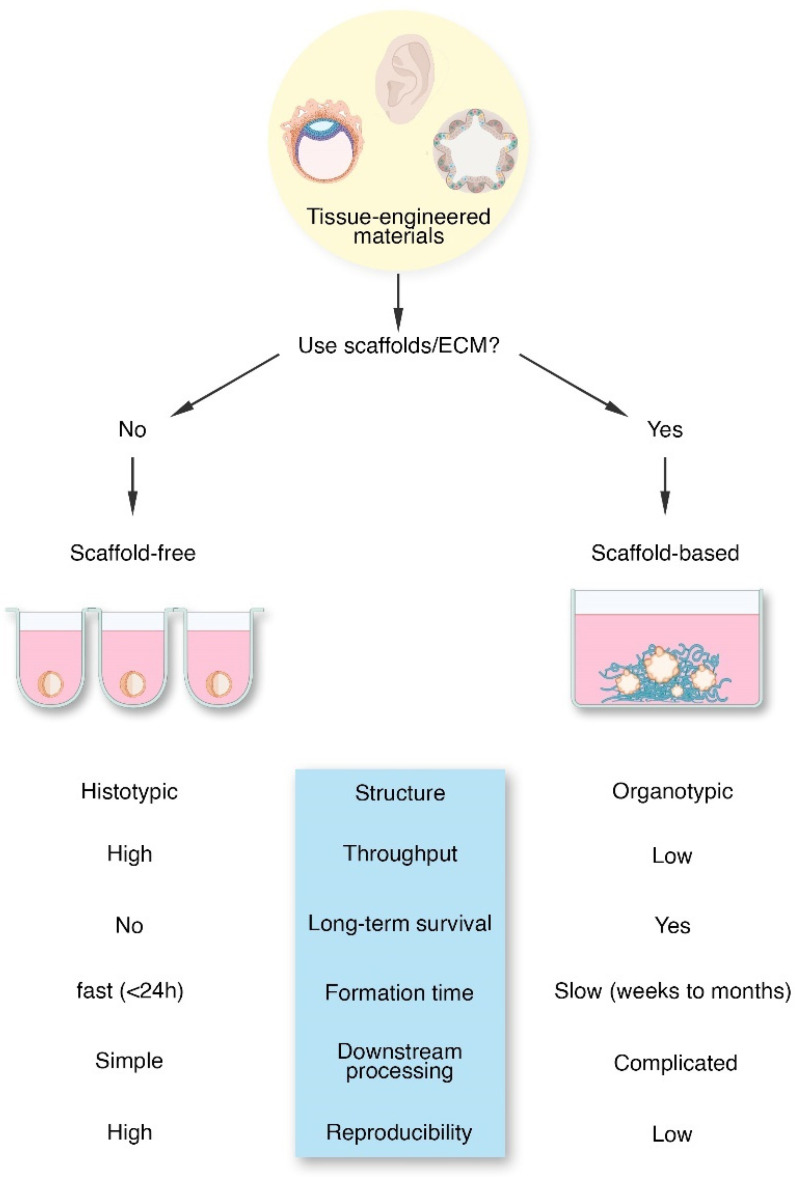
The tissue engineering dichotomy. In tissue engineering, there are two major ways to create 3-dimensional cell aggregates, tissues, or organs, depending on the use of scaffolding materials, such as ECM and ECM-mimetic substances. Scaffold-free techniques often require ultra-low adherent surfaces or methods that would lead to forced-aggregation of cells into spheroids. In contrast, scaffold-based techniques use sold scaffolds (hydrogels, ECM, or synthetic polymers) to guide tissue or organ formation in vitro. As highlighted here, both techniques have pros and cons and the decision often lies with what is more relevant for a specific scenario.

We previously discussed the functions of the ECM and its role as an indispensable biomaterial for tissue engineering. In tissue engineering, the decision whether to use ECM and ECM-mimetic materials or not leads to a dichotomy—to scaffold or not to scaffold? (Figure 3) There is no correct answer to this question as both techniques have advantages and disadvantages. Though, due to the inherent culture differences, tissue-engineered products show significant disparity. The scaffold-based approach uses an exogenous material as a substitute for the native organ ECM. This allows for manipulation of the mechanical and chemical aspects of the cell’s extracellular environment, as well as greater structural integrity for large tissue regeneration [133]. On the other hand, the scaffold-free approach often forms tissues by forcing cells to aggregate into small spheres, hence the term “spheroids”, which can develop an endogenous ECM [8]. This approach has several benefits including high initial cell density, rapid formation, and a self-organized tissue-like structure [8].

### 3.1. Scaffold-Free Tissue Engineering

Scaffold-free tissue engineering encompasses efforts to create a variety of tissues without the aid of scaffolding materials. Several techniques encompass this field including cell sheet and spheroid technology. Spheroids are multicellular aggregates often ranging in diameter from 50–1000 μm [134] and can be formed using various techniques, including hanging-drop cultures, microfluidic devices, agitation-based bioreactors, or cultures on low-adhesive materials [134,135,136]. Though these techniques are different, they share fundamentally similar process, that is, the forced aggregation of cells to create cell clumps. By this, tissue-engineered spheroids have high culture efficiency, high cell density, and 3D cell–cell interactions which are often achieved in less than 24 h [8,135]. Due to its simplicity and reproducibility, scaffold-free culture techniques allow for automated production of hundreds to thousands of spheroids within 24 h, which is highly advantageous for high-throughput drug screening and other applications [137]. Spheroids naturally form from a high cell seeding density, which may require months of culture with scaffold-based approaches [8,138]. As cells are forced to aggregate in spheroid culture, they are provided with 3D interactions with other cells, which promotes self-organization [8]. These self-organized cell aggregates have the potential to mimic tissue properties. In Table 3, we have outlined several research and applications of scaffold-free tissue-engineered materials in therapeutics, disease modeling, and studying developmental biology.

However, spheroid formation causes a gradient of nutrients, providing less nutrients to inner cells compared to cells closer to the edge of spheroids [134]. This concentration gradient in the aggregate mimics the scale of concentration gradients during embryogenesis but leads to necrosis for spheroids larger than a few hundred microns in diameter [135]. Because the inner cells receive less nutrients, even smaller spheroids experience inner core necrosis when cultured under hypoxia or other strenuous conditions [135]. This concentration gradient and core necrosis characteristics shown in solid tumors make spheroids a viable 3D model for cancer [149,150,151]. Downstream applications of spheroids include using them as building blocks to form larger tissues, though there is evidence that the mechanical properties of the individual scaffold-free tissues are inferior, leading to cell damage during this process [8]. Finally, scaffold-free cultures at least initially lack connection to ECM proteins, which can induce cell death via anoikis [152].

Although spheroids are not supplemented with exogeneous ECM, cultured spheroids have been shown to secrete ECM proteins. Early studies on human glioma and thyroid cancer spheroids demonstrate the endogenous production of fibronectin, laminin, collagen, and glycosaminoglycans, and were comparable to in vivo tumors [153]. There are several factors affecting the production of ECM in spheroids, including the age of cell source [154] and the culture method (such as hypoxia) [155]. However, certain cells are more susceptible to the effects of low ECM levels, such as halted growth, senescence, and even cell death. Specifically, fibroblast spheroids have been shown to have limited growth in scaffold-free cultures [156,157,158]. Once in spheroids, the growth of fibroblasts is arrested, leading to quiescence [158] and necrotic programmed cell death (nemosis) [159]. The major purported signaling pathway associated with this is the Hippo signaling which senses cell-to-cell contacts as an anti-proliferative signal resulting in the inhibition of YAP/TAZ pro-mitogenic transcriptional roles [160,161]. More recent attempts to increase ECM synthesis via a doxycycline-inducible nuclear-localized TAZ has been shown to promote 3D growth in fibroblast spheroids but failed to promote the production of collagens and fibronectin [161]. However, results show that ECM-based signaling is a negative upstream regulator of Hippo [162,163]. Moreover, Hippo is regulated via adhesion to fibronectin which then activates the focal adhesion kinase (FAK) signaling axis, leading to activation and nuclear translocation of YAP [164].

### 3.2. Scaffold-Based Tissue Engineering

Compared to scaffold-free methods, scaffold-based tissue engineering encompasses a variety of methods to create tissues with features mimicking the native solid ECM—either by natural or synthetic scaffolds. Scaffold-based tissue engineering generally seeks to create a solid superstructure that can induce the directed growth of cells towards organ-like formation. Solid scaffolds offer many benefits, such as being customizable to influence cell patterning and presenting biochemical signals in a temporally and spatially controlled manner [165]. It is well established that scaffold-based tissue engineering enhances cell survival and differentiation [166], as shown in bone [167], skin [168], cancer [169], cardiac [170], or muscle tissue engineering [171]. Moreover, using ECM, especially the BME in the production of organoids, is a primary focus in the field. We have compiled some of the most compelling organoid research in Table 4 and their applications in disease modeling. However, cell seeding in solid scaffolds is often problematic. When cells are introduced to solid scaffolds, they show superficial penetration into the scaffold and slow migration to the interior [8,172,173]. Though, cell penetration can be remedied by introducing cells prior to scaffold gelling or solidification. Although reseeding in decellularized tissues has progressed, the integration of a functional vasculature is still a major problem. Without a functional vasculature, oxygen and nutrients do not readily diffuse into engineered tissues. This limits the size and viability of engineered tissues, preventing translational application [174].

Cell-adherent hydrogels are widely used as scaffolds in tissue engineering. As described earlier, these biomaterials have similar functions to the native ECM [13]. They can be formed using various techniques starting from a liquid phase, which allows tissue engineers to homogenously incorporate cells into a supporting scaffold [8]. These cell-laden hydrogels can then be spatially arranged with a 3D bioprinter, allowing tissue engineers to mimic in vivo organization of the ECM [186] and increase throughput [187]. Cell aggregates created using scaffold-free tissue engineering techniques can also be embedded into hydrogel scaffolds, a common technique for creating organ-mimetic cell aggregates or organoids [188].

However, hydrogel-based tissue engineering also encounters challenges. Hydrogels limit the diffusion of nutrients, which decreases the nutrients available to organoids at the core of the hydrogel domes compared to those close to liquid media [135]. The hydrogels themselves can also induce overall heterogeneity in tissue engineering through their composition, as demonstrated by the batch-to-batch variability in Matrigel scaffolds [189] and variability in composition of decellularized organ-derived ECM [190]. Recent developments in biomaterial science and engineering, as reviewed previously [see Section 2.4], could present some solutions to this problem. Aside from heterogeneity, the stiffness of hydrogels can inhibit cell migration and spreading, preventing cells from readily organizing into tissues [8]. We then posit that this is the prime reason why scaffold-based tissue engineering takes a longer time than scaffold-free cultures to reach optimal levels of confluency. Moreover, organoid extraction from solid scaffolds is cumbersome [191]. Aside from these, downstream processing in these cultures is impeded by the solid scaffolds which are difficult to remove [105]. Lastly, certain hydrogels, such as polyacrylamides, pose cytotoxicity in 3D culture [192], thus cytotoxicity studies are needed in new biomaterials for 3D culture. In summary, scaffold-based tissue engineering is highly biologically relevant due to the presence of organ-like ECM though problems in consistency and reproducibility which needs to be addressed.

## 4. Hybrid Tissue Engineering and Use of ECM

**Figure 4:**
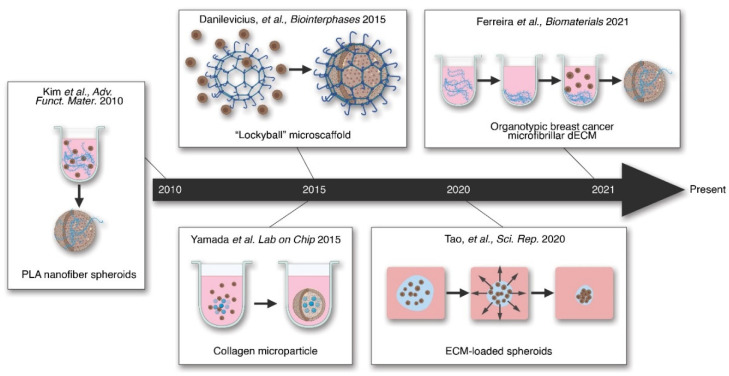
Timeline of key hybrid tissue engineering methods. Hybrid tissue engineering methods incorporate the ECM or similar scaffolds into the cell aggregates. These confer the aggregates improved tissue properties. Recently, a hybrid culture has been shown to allow organ-like formation in spheroids [193].

Recent efforts in tissue engineering attempt to combine aspects of scaffold-based and scaffold-free techniques. This hybrid tissue engineering technique offers benefits of both scaffold-based and scaffold-free culture. It was recently argued that hybrid tissue engineering may become a ‘disruptive’ technology, enabling tissue engineering to overcome the barriers to clinical application [8]. Novel hybrid tissue engineering methodologies are illustrated in Figure 4.

One innovative example of hybrid tissue engineering is shown by the integration of spheroids into a microscaffold referred to as the “lockyball” [194]. These microscale lockyballs provided an exogenous structure to facilitate the fusion of multiple spheroids into a larger, customized structure. This innovation provides a platform to increase the size and control the shape and mechanical properties of the tissue, while simultaneously providing high cell density. The microstructure also provides needed mechanical support for regenerative medicine applications [195].

Another method of hybridized scaffold and scaffold-free tissue engineering techniques involves adding ECM-like materials during the formation and maintenance of spheroids. An early example of this method incorporated degradable microspheres which releases retinoic acid, a signaling molecule, into embryonic stem cell spheroids [196]. This synergistic approach mimicked the signaling functions of native ECM and enhanced the differentiation of the stem cells into embryoid bodies. Kim and colleagues expanded on this novel approach by integrating functionalized poly(L-lactic acid) (PLA) nanofilaments into mesenchymal stem cell (MSC) spheroids during the initial culture step [197]. These composite spheroids were exposed to adipogenic induction medium and have increased expression of adipocyte markers compared to spheroids without the ECM-like nanofilaments. These findings give evidence that the addition of ECM-like particles can increase the impact of organogenesis-inducing signaling factors. Yamada and colleagues expanded on these findings through a similar integration of cell-sized collagen microparticles into primary hepatocyte spheroids [198]. They demonstrated the combined consistency inherent in spheroid culture, while also showing an increased expression of hepatocyte-specific function, as measured by the production of albumin and other proteins. Their results further demonstrate the potency of ECM to increase organotypic function of scaffold-free derived spheroids. Tao and colleagues added to these results showing enhanced hepatocyte function in a spheroid culture method where Matrigel was precipitated into hepatocytes forming hepatic spheroids [199]. Importantly, they found that the addition of basement membrane proteins increased the formation of cell polarity features critical to hepatocyte function. This observation is consistent with the role of the basement membrane in contributing to cell polarity in vivo [69].

Additionally, a recent report showed that breast cancer tumor spheroids could be improved by the addition of decellularized breast tissue microfibers [193]. This report detailed incorporating tissue-specific ECM from porcine breast tissues during the initial forced aggregation step. The integration of tissue-specific ECM fibers resulted in a disease model that better replicated the cancer phenotype compared to spheroids without the fragments and exhibited an increased specific resistance to chemotherapy regimens. These results highlight the vital role of ECM in modeling different disease states. In summary, these results collectively show the potential of hybrid tissue engineering to better replicate specific organotypic features of disease models while maintaining the robust consistency and reproducibility of scaffold-free culture.

## 5. Conclusions and Future Outlook

The study of the ECM has advanced tissue engineering and regenerative medicine in countless ways. Being nature’s original bioscaffold, a plethora of information can be discovered from understanding the ECM. The use of decellularized materials from organs provide the most natural source for ECM for use in tissue engineering. The most prevalent problem in decellularized materials is the animal-to-animal variability in ECM composition which leaves no clear chemical definition. This variability also leads to problems of reproducibility in some tissue engineering experiments.

A potential answer to this problem is the creation of novel ECM-like polymers. Recent advancements in biomaterials science and engineering show specific features of the ECM can be mimicked by biocompatible polymers. Though, these materials are far from perfect; most only recapitulate one feature of the ECM, for example, RGD-functionalized PEGDA polymers can recapitulate cell adhesion but not degradation and remodeling. Thus, we view that further advancements in this field will provide novel materials that mimic ECM with higher fidelity. We believe that the development of chimeric biomimetic scaffolds—synthetic polymers with multiple biologically functional parts—will provide chemically defined and tunable materials for tissue engineering. Functionalization of these polymers to closely mimic the biological ECM is necessary. A perfect biomaterial is one that would show the pleiotropic effects of the ECM ranging from cell scaffolding to cell signaling for survival. To achieve this, first, effective mining of current literature on which motifs translate to biological responses is necessary and, second, the integration of these motifs in bio-functional polymers to provide us a perfect ECM mimetic. Another possible approach to achieve a perfect ECM mimic could involve multiple polymers with various functional groups. By this, the ECM nature of having multiple polymers—proteins and glycans—is being replicated.

Current literature has expanded the role of the ECM as a dichotomizing force in tissue engineering, where the decision to use and not use ECM or ECM-like materials determines the type of tissue engineering being performed. However, by building a middle ground, we can synergize these disparate tissue engineering techniques. A recent account shows that scaffold-free production of 3D in vitro tumor models was improved by incorporation of pelleted decellularized ECM into ultra-low adhesion (ULA) surface prior to cell aggregation [193]. This technology was also used to recapitulate organotypic features of breast cancers which have not been shown using prior methods. Due to its similarity to scaffold-free technique, the production of reproducible spheroids in a high-throughput manner was attained. Further studies on novel ways of incorporating ECM into scaffold-free cultures are currently needed. Moreover, use of biomimetic synthetic ECM in a synergistic culture is yet to be done. We posit that another way to incorporate ECM into scaffold-free cultures is to take advantage of ECM solubility. Research shows that the soluble form of ECM protein tropoelastin drives mesenchymal stem cell proliferation and differentiation [200]. Several studies indicate that urea-extracted soluble ECM proteins enhance the differentiation of mesenchymal stem cells in knee meniscus and tendon tissue engineering [201,202]. Importantly, the soluble ECM derived from respective tissues enhances specificity of cell differentiation and increases the strength of tissue-engineered tendons [202]. Researchers theorize that soluble ECM proteins signal cell differentiation and specific remodeling of the surrounding ECM scaffold [202]. Another group found that conditioned media containing soluble laminin enhanced lung ECM recellularization, increasing cell attachment by more than two-fold [203]. The implications of this body of research are important, as a soluble ECM is more easily delivered, and the maintenance of soluble ECM cultures is simplified and can conform to good manufacturing practices (GMP) more easily.

In summary, the use of ECM has led to a division in tissue engineering techniques. In most cases, an almost existential question arises—“to scaffold or not to scaffold?”. Scaffolding is an essential part of tissue engineering as it is in organs, and a lack of it leads to less organotypic modeling. Biomimetic materials for the ECM are effective for use in tissue engineering. However, further research in creating biomaterials that mimic the plethora of ECM effects in the cells is needed. We view that ECM plays a huge role in bridging the disparity in tissue engineering techniques. Precise methodical approaches are needed to effectively deliver ECM into scaffold-free culture. We view that progress in both material science and novel hybrid tissue engineering can hand-in-hand improve the way that scientists and engineers create 3D models, leading to robust culture techniques in the future.

## 6. Glossary

**Decellularization:** the process of removing cells from biological tissues and organs via mechanical stress, detergents, or other chemical agents. Decellularized tissues could be re-cellularized or digested into extracellular matrix-derived hydrogels.

**Extracellular Matrix (ECM):** the acellular component of the organs and tissues. It provides structural support to tissues and anchorage to cells, and modulates mechanotransduction pathways, cell death, and cell fate, among others. In tissue engineering, the most commonly utilized ECM is the basement membrane extract (BME), often derived from murine sarcoma. BME often come in tradenames, such as Matrigel, Cultrex, or Geltrex. Organ ECM could also be used in tissue engineering. These materials are derived through the process of decellularization.

**Organoid:** a 3-dimensional cell aggregate that mimics biological organs. They are organized similar to organ complexity. Often, these are created from stem cells—induced pluripotent stem cells, embryonic stem cells, and even adult progenitor stem cells. Most common patient-derived organoids are created using adult progenitor stem cells specific to the organ epithelium. These then mimic the complexity of the organ or cancer epithelium (epithelial organoids).

**Scaffold-based tissue engineering:** techniques encompassing the formation of tissues and organs in vitro with the use of scaffolding materials (ECM, synthetic scaffolds, etc.) to provide constructive and instructive signals to the cells.

**Scaffold-free tissue engineering:** techniques encompassing the *de novo* formation and self-assembly of tissue and organ-like structures without the aid of scaffolding materials (ECM or bioscaffolds). This technique includes forced aggregation, hanging drops, bioreactor cultures, and cell sheet technologies among others.

**Spheroid:** a 3-dimensional cell aggregate that mimics biological tissues. They often come in simple, homogeneous structures formed by mature, differentiated cells.

## Figures and Tables

**Table 2:** Peptide sequences used in functionalizing synthetic polymers.

Peptide	Role/Effect	Polymer	Citation
RGD	Adhesion	PEG	Matsuda, et al., 1989 [132] Hern and Hubbell, 1998 [119]
PQ	Adhesion, degradation remodeling	PEG	Lutolf, et al., 2003 [127]
**Degradation rate variable:** GPQGIWG (slow), VPMSMRGG (fast)	Cell migration and Proliferation (with fast construct)	PEG	Patterson and Hubbell, 2010 [128]
**Cleavage linkers:** CGPQGIAGQGCR (NCD-CR) KCGPQGIAGQCK (NCD-KK) KCGPQGIAGQACK (NCD-KAK) KCDGVPMSMRGGCK (HD)	Degradable crosslinkers	Dextran	Liu, et al., 2021 [116]

**Table 3:** Applications of scaffold-free tissue engineering.

Application	Culture Method	Application Method/Results
Therapeutics	Autologous skeletal muscle tissue sheet	Transplantation to the patient epicardium, improved cardiac disease symptoms [139]
	Suspension culture of cartilaginous spheroids from human iPSC	Implantation into tibial fractures in nude mice, limited induction of bone remodeling [140]
	Suspension culture of primary porcine hepatocyte spheroids	Implantation into extracorporeal device, improved outcomes in a porcine acute liver failure model [141]
	Bio-printed trachea from suspension culture of primary rat chondrocyte, mesenchymal stem cells, and lung epithelial cells	Transplantation into rats show vasculogenesis and chondrogenesis [142]
Disease Modeling	Suspension culture of non-small-cell lung cancer spheroids	Genome-wide CRISPR high-throughput drug screen against 3D cancer growth [143]
	Hanging-drop culture of stable and tumor-derived, murine cells to form mammary spheroids	Studying neoplastic progression in spheroids with high consistency and reproducibility [144]
	Suspension spheroid co-culture of HepaRG and primary-derived hepatic stellate cells	Identification of acetaminophen as driver of stellate activation in liver fibrosis [145]
	Suspension culture-based differentiation of iPSCs into 3D neuro-spheres from Alzheimer’s disease patients	Drug screen of spheroids showed a patient-specific response to a specific drug [146]
Developmental & Stem cell studies	Suspension culture of human hPSC, differentiated to cardiomyocytes	Characterized the effects of spheroid culture and cell density on cardiomyocyte differentiation [147]
	“Lungosphere” suspension culture from primary-derived murine lung epithelial cells	Characterization of lung epithelial stem cells, validation of novel assay to separate lung epithelial stem cells [148]

**Table 4:** Scaffold-based organoids and their applications in disease modeling.

Organ Type	Disease Applications
Brain	**Autism**—Patient-derived iPSC developed into neural organoids show overproduction of GABAergic inhibitory neurons [175]. **Microcephaly**—Patient-derived human iPSC cerebral organoids model microcephaly via loss of the CDK5RAP2 protein [176]. **Viral infection**—Human neural stem cell organoids infected with Zika virus exhibited decreased size and increased death of brain cells [177]. **Cancer**—Human glioblastoma-derived organoids maintain tumorigenic potential and heterogeneity [178].
GI tract	**Ulcerative colitis**—Human epithelial organoid cultures from UC patients exhibited differences from non-UC patient organoids in expression of genes associated with microbial defense, secretion, absorption, and gastric phenotype [179]. **Cancer**—CRISPR/Cas9 was used to modify 4 commonly mutated colorectal cancer genes in human intestinal stem cells. The organoids produced were xenotransplanted into mice and grew as tumors with features of invasive carcinoma [180].
	**Cystic Fibrosis**—The drug response in primary rectal organoids from CF patients can be used to predict the patient’s specific drug response [181].
Liver	**Liver failure**—Human 3D bioprinted hepatorganoids transplanted into Fah-deficient mice rescued liver function and improved survivability of mice [182].
Lung	**Cancer**—Patient-derived organoids formed from human lung adenocarcinoma cells retained the architecture and gene expression of the tumors they were derived from [183]. **Pulmonary fibrosis**—Human pluripotent stem cells were used to develop alveolar epithelial organoids of differentiated cells. TGF-β1 treatment induced fibrotic changes [184].
Pancreas	**Cancer**—Primary human pancreatic adenocarcinoma organoids retain heterogeneity and histoarchitecture of parent tumor as well as physiological changes specific to the patient of origin [185].

## Data Availability

No new data were created or analyzed in this study. Data sharing is not applicable to this article.
